# Impacts of LULC and climate changes on hydropower generation and development: A systematic review

**DOI:** 10.1016/j.heliyon.2023.e21247

**Published:** 2023-10-27

**Authors:** Emmanuel Kekle Ahialey, Amos T. Kabo–Bah, Samuel Gyamfi

**Affiliations:** a Department of Renewable Energy Engineering, School of Energy, University of Energy and Natural Resources (UENR), P. O. Box 214. Sunyani, Ghana; b Regional Center for Energy and Environmental Sustainability (RCEES), University of Energy and Natural Resources (UENR), P. O. Box 214. Sunyani, Ghana; c Department of Civil and Environmental Engineering, University of Energy and Natural Resources (UENR), Sunyani, Ghana. P. O. Box 214. Sunyani, Ghana; d School of Energy, University of Energy and Natural Resources (UENR), Sunyani, Ghana. P. O. Box 214. Sunyani, Ghana

**Keywords:** Climate change, land use land cover (LULC), Streamflow, Hydropower generation, Hydropower development

## Abstract

There is a growing concern on a global scale that the world should transition towards the utilisation of energy-efficient technologies. Hydropower plays a very significant part in the fight against climate change, and as a result, it lessens the impact that climate changewill have on our ability to achieve the Sustainable Development Goals (SDGs). Both the effectiveness of hydropower generation and the amount of streamflow are impacted by climate change as well as land use and land cover (LULC). Accordingly, the purpose of this study is to conduct a literature review on the topic of the past and future effects of climate, land use, and land cover changes on hydropower generation. This review will be based on the entries found in a number of reliable databases. A systematic literature review was carried out to analyse how LULC and climate change will affect hydropower generation and development. The research was based on 158 pieces of relevant literature that had been reviewed by experts and indexed in Scopus, Google Scholar, and ScienceDirect. The review was carried out to determine three goals in mind: the impact of climate change on hydropower generation and development; the impact of climate change on streamflow; and the combined impact of changes in climate and changes in LULC on hydropower. The findings bring to light the primary factors contributing to climate change as well as shifts in LULC which are essential to the generation of hydropower on all scales. The study identifies factors such as precipitation, temperature, floods, and droughts as examples of climate change. Deforestation, afforestation, and urbanisation are identified as the primary causes of changes in LULC over the past several decades. These changes have a negative impact on the generation and development of hydropower.

## 1 introduction

The generation of electricity through the burning of fossil fuels has been a major contributor to the release of gases that contribute to global warming [1]. These gasessuch as coal, gas, as well as light crude oil (LCO) are hastening the process of climate change (CC) and global warming [1]. The generation of energy from fossil fuels is widely held by professionals to be detrimental to the environment over time [2]. It is also regarded as the primary source of anthropogenic greenhouse gas (GHG) emissions, which is a major factor in the progression of CC [2].The emission of GHGs continueto rise, primarily due to the unsustainable energy use, land-use change, lifestyles, patterns of consumption and production [3]. [4] found out that the number of GHGs produced from the use of coal would have been between 80 and 100mmt of carbon if the energy that was produced from hydropower in 2019 alone had come from the combustion of coal. As a result of the continuous release of GHGs into the atmosphere, the global surface temperature has seen an astronomical increase especially in the 21st century. This happens across regions, between and within countries, and among individuals [ [3,5]]

The Intergovernmental Panel on Climate Change [ [3,5]] stated that the global surface temperature in the first two decades of the 21st century (2001–2020) was 0.99 °C higher than 1850–1900. In the same publication, every region of the world is projected to see an increase in multiple changes in weather patterns.According to projections in Ref. [6], if the world's temperature rises by 1.5 °C in the near future, the world will be forced to deal with increases in several climate threats that cannot be avoided and will pose multiple risks to ecosystems as well as humans. Beyond the year 2040, the resultant effects of re-solves of hazards for both natural and artificial systems, will depend on the level of global warming [6]. In addition [6], report that the degree of danger can be determined by simultaneous developments in susceptibility and exposure in the short term as well as the degree of social and economic growth as well as flexibility.

These new climatic patterns, are likely to have a negative impact on the generation and development of hydropower. Evidences already abound as [7]report about erratic patterns of rainfall and extreme weather events such as floods, droughts, heatwaves and tropical cyclones have become more intensified in many countries across the globe.Additionally, the sixth assessment report of IPCC projected a reduction in water availability, which could affect water supplies needed for hydropower use [5]. It further projected regions in Africa, Australasia, Europe and North America to have increases in hydrological droughts [5]. Experts have therefore proposed limiting the global warming and this involves a rapid, deep and immediate reduction in the GHGs [3]. According to Ref. [8], there will be a need for 850 GW (GW) of new hydropower capacityin order to keep the rise in temperature above the pre-industrial level to less than 2 °C by the year 2050.

At this juncture, the utilisation of clean sources of energy (hydropower, wind, solar, geothermal, and nuclear) is very important especially as the world is working towards the accomplishment of the Sustainable Development Goals (SDGs) [1]. It is also crucial to have a policy change especially in the face of the new climatic patterns so as to prevent the slowdown of hydropower development.

### 1.1 relation between hydropower plants and the emission of GHGs

The major greenhouse gases are carbon dioxide (CO_2_), methane (CH_4_) andnitrous oxide (N_2_O). Studies have shown that the aforementioned gases are emitted from terrestrial ecosystems, anthropogenic and naturalaquatic sources [9,10]. Above studies, have established the fact that, GHGs are also emitted from all freshwater systems such as lakes, rivers, estuaries, wetlands, seasonal flooded zones and reservoirs.

Clean energy technologies, such as hydropower, are known to have made contribution to the reduction of GHG emissions and to the security of the energy supply. For some decades now, there has been a division as to either hydropower plants emits GHGs or not. Majority of nine articles published/cited in Ref. [11]give credence to the fact thathydropower plants in Brazil's Amazonianemit GHGs Ref [eg. [[12], [13], [14], [15], [16], [17], [18]]]. This calls for a thorough research in the Amazonian and other regions to ascertain the relationship between hydropower plants and GHG emissions.

Notwithstanding, the World Energy Council (WEC), stated that the CO_2_ emissions per GWh are 3–4 t for hydropower run-of the river, and 10–33 t for hydropower with a reservoir. Additionally, they stated that the values above are about 100 times less than the emissions from conventional thermal power [19]. The World Bank report alsoheld the view that GHG emissions seem to be relatively small for the majority of reservoirs [20]. No matter the case [21], held the view that the quantities of GHG emissions from hydropower are always much lower than those from thermal power. In general, hydropower is a source of energy that produces few GHG emissions.

### 1.2 contribution of Hydropowerto low-carbon energy

Hydropower is currently the largest source of low-carbon energy globallywhich meets approximately 16 % of the demand for electricity all over the world [8]. It also contributes towards the mitigation of global warming [22]. Since 2015, the total hydroelectric capacity of the world has been growing at an annualised rate of 2.1 % on average [23].

The majority of power supply over the decades in most advanced economies such as Norway, Canada, Switzerland and Austria, is from hydropower [24]. In 2021 alone, the hydropower installed capacity reached 1360 GWwhich is an addition of 1.9 % of the 2020 figure [25]. Despite the enormous contribution of hydropower, the untapped hydropower potential in the various regions remains a key policy concern to expanding electricity access and meeting growing demand in Africa and Asia [24].

**Fig. 1:**
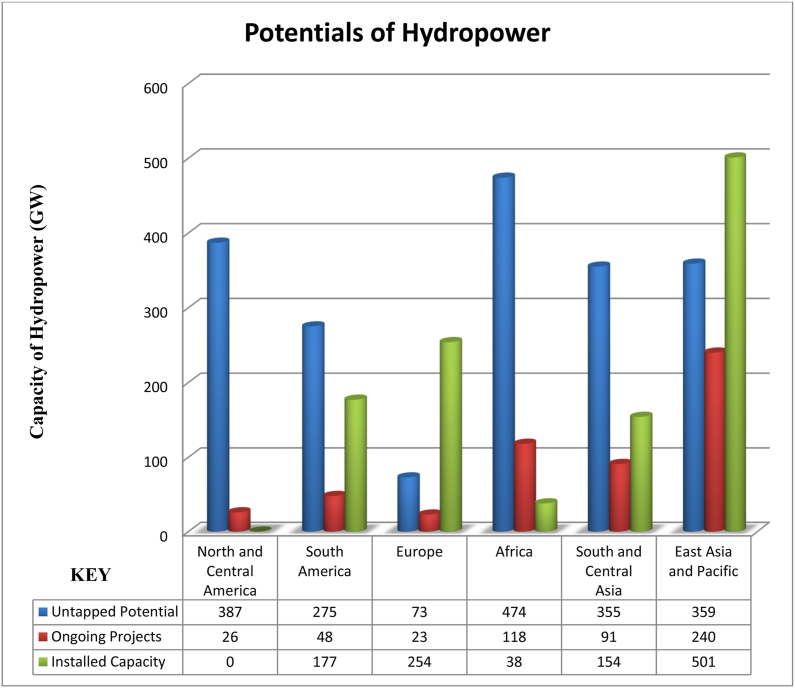
Potentials of hydropower across the regions.

Fig. 1 shows the global hydropower potential by regions. It however excludes the pumped storage hydropower potentials.

Source [25].

### 1.3 Organization of the review

Previous reviews conducted by Refs. [26,27] investigated the impact that CCand its variability has had on the production of energy around the world and organised their findings in accordance with geographical regions. Additionally, the global review conducted by Ref. [28] investigated the connection between rising global temperatures and the use of hydropower industry by offering a focused and in-depth investigation of the consequences CCwill have on the hydropower industry.

Studies carried out by Refs. [[29], [30], [31], [32], [33]], dealt with the impact of CC on hydropower. Also, previous reviews by Refs. [[26], [27], [28],[34], [35], [36]], have provided an insight into general repercussions of CC on the hydropower sector. Noneof the previous papers reviewed the combined impact of CC and LULC on hydropower and specifically highlighted the key drivers of CCand LULC.As a result, we conducted an in-depth analysis of the impacts that CC and LULC have on the production and advancement of hydropower. In addition to this, we gave an overview of key drivers that makes a significant contribution in the generation and development of hydropower.

**Fig. 2:**
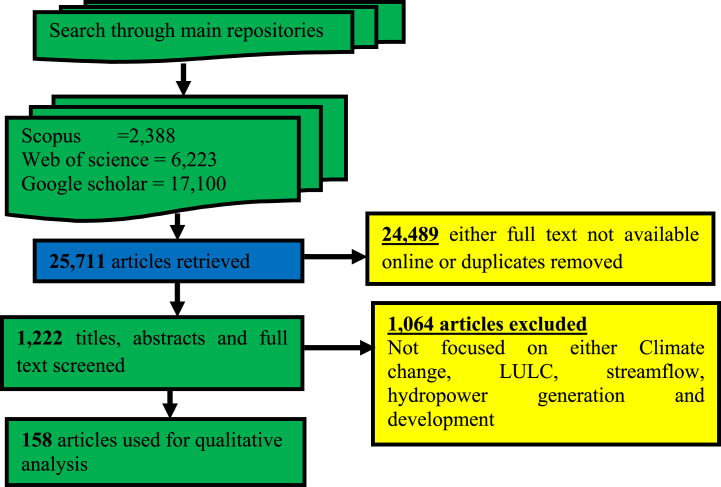
Process flow diagram for the selection of studies using PRISMA

This review papercontains three sections. In the **first section**, we discussed the relationship between hydropower and GHGs, contributions of hydropower to low carbon energyand highlighted the potentials of each region. The **second section**looked at the literature review methods that were applied, as well as the design of the research. It highlights all regions (Fig. 2) that are likely to experience potential changes in the future due to CC, LULC, and the combinationofCC and LULC.The outcomes as well as sub-sequent discussion are presented in the **third section.** This includes important summaries of key findings from existing studies by region (Table 1, Table 2, Table 3). Additionally, key drivers of CC and LULC that impact hydropower generation were included. We finally highlighted the strategies to mitigating the impact of CC on hydropower generation. This was followed by the study's summary and conclusions presented in the **fourth section**.

**Table 1:** A Summary of Impacts of CC on the generation and development of hydropower.

Region	**Reference**	**Main Purpose of the study**	**Method/Model Used**	**Climate Scenario/GCM**	**Main Outcomes Identified**
North and Central America	[37]	Climate change impact on water supply and hydropower generation potential in Northern Manitoba	1. HYPE (Hydrological Predictions for the Environment) 2. WATFLOOD and 3. HEC-HMS and 4. An operations model, MODSIM-DSS	No data	1. The forebay will expand during the spring and summer but will contract during the winter and fall 2. It is anticipated that the potential for hydropower generation will increase during the spring (season of flooding). 3. A decrease in the amount of water that flows into reservoirs and the amount of potential hydropower that can be generated
	[38]	Implications of hydropower variability from climate change for a future, highly-renewable electric grid in California	CanESM2, CNRM-CM5, HadGEM2-ES, MIROC5, HadGEM2-ES	RCP8.5	1.CC impact on hydropower can increase greenhouse gas emissions up to 8.1 % due to increased spillage of reservoir inflow reducing hydropower generation. 2.Increases in dispatchable power plant capacity of +2.1 to +6.3%and decreases in the number of start-up events per power plant unit up to 3.1 %.
	[39]	Climate-change impacts on water resources and hydropower potential in the Upper Colorado River Basin	WARMF hydrologic model. 3 GCMs	2 SRES scenarios: A2 and B1	1. Precipitation projections vary up to 16 %; but flow projections up to 50 %. 2. Projections of increase in temperature at low elevations withextreme seasonality at high elevations. 3.Projections of a 60%decline in precipitation at lower elevations and a 74 % increase at high elevations 3. Overall decrease in annual flow using the A2 scenario.
East Asia Pacific	[40]	Potential Impact of Climate Change on Hydropower Generation in Southern Taiwan	GWLF simulations from four GCMs.	Scenarios A2 and B2	1.During the dry season, the range of variation in river discharge was −26%-15 %, and during the wet season, the range was -10-82 %. 2.The consequences of climate change may have an effect on Taiwan's hydrology as well as its ability to generate hydroelectric power.
	[41]	Future hydropower generation prediction of large-scale reservoirs in the upper Yangtze River basin under climate change	Canadian Earth System Model (CanESM2)	RCPs2.6, 4.5 and 8.5	1. No significant increase or decrease in the trend of hydropower generation in the future, according to the RCP2.6 scenario. 2. The generation of hydropower exhibits an upward trend. 3. The growth of hydropower generation is highly susceptible to changing climate impact.
	[42]	Climate change impacts on Three Gorges Reservoir impoundment and hydropower generation	SWAT	RCPs 2.6, 4.5 and 8.5	1.Precipitation inthe basin is expected to increase 2.The projected mean annual inflow and hydropower generation of the TGR will increase by 3.3–5.6 % and 0.9–2.3 % in 2040–2065, 7.9%–15.2 % and 5.2–8.1 % in 2080–2099 respectively 3.The inter-annual variation ofpower generation will increase especially in the dry season. 4.The reservoir performance is highly sensitive to thechanges in the seasonal distribution and extremes of streamflow. 5.The utilisation rate of water resources underprojected extreme streamflow is expected to decrease
	[43]	Impacts of climate change on hydropower generation in China.	Econometric Model	RCPs 2.6, 4.5 and 8.5	1.At the national level,the influences of climatic factors on hydropower generation shows, the elasticity coefficients of rainfall, heating degree day (HDD), cooling degree day (CDD) and sunshine duration are 0.081, −0.016, 0.089 and −0.043 respectively. 2. The impacts ofclimatic factors on the hydropower generation in the northern and southern regions of China are different. 3. The effect of rainfall onthe hydropower generation is significant in the southern regions, but not in the northern region. 4. The CDD has a significant effect onboth the northern and southern regions, and the latter is greater (0.136 %). 5. The impact of HDD on the northern area is significant, while its influence on the southern area is not significant. 6. The influence of sunshine duration is not significant in any region inChina.
	[44]	Evaluation effect climate parameters change on hydropower production and energy demand by RCPs scenarios and the Developed Pathfinder (DPA) algorithm.	HadCM3 model	RCPs 2.6 and 8.5	1.The forecasting of the climaticparameters showed that in the next 25 years, the temperature will increase and rainfall will decrease. 2. Due to the impact ofpopulation, Gross Domestic Product (GDP), and rising temperatures, society would face an increasingtrend of energy demand. 3. Due to the increase inthe consumption of cooling devices the demand for energy will be higher in some seasons.
	[45]	Evident response of future hydropower generation to climate change	The variable infiltration capacity model is coupled with global climate models	RCPs 2.6, 4.5 and 8.5	1. Thefuture reservoir inflow and hydropower outputsignificantly differ from the historical ones. 2. Compared with the historical period, the reservoir inflow and hydropower output under all threescenarios show larger fluctuations 3. Overall increase in inflow and hydropower output is expected infuture wet years, but large decrease is found in dry years and no obviouschange exists in normal years. 4. Future hydropowerin dry years would have higher vulnerability with lower reliabilityand resiliency.
	[46]	Forecast of the hydropower generation under influence of climate change based on RCPs and Developed Crow Search Optimization Algorithm.	GFDLCM3 model	RCPs 2.6, 4.5 and 8.5	The prediction of CC under RCPs indicates that the average annualpower generation will decrease under RCPs 2.6 4.5 and 8.5 about 10.74 %, 16.38 % and22.25 % respectively. 2. The average annual power generation under RCPs 2.6, 4.5, and8.5 by 2050 are 740.33 MW, 603.12 MW, and 585.77 MW, respectively.
	[22]	Assessment of the impact of climate change on hydropower potential in the Nanliujiang River basin of China	The variable infiltration capacity (VIC) hydrological model	RCPs 2.6, 4.5 and 8.5	1. Varying degrees of future changes in river discharge result in an increase of 7.7 %–15.6 % in hydropower potentials.
	[47]	Projection of climate change impacts on hydropower in the source region of the Yangtze River based on CMIP6	Variable infiltration capacity model (VIC)	SSP2-4.5 and SSP5-8.5 scenarios.	1.The precipitation in the Yalong River basin showed an increasing trend (12.38 mm/10a, 25.65 mm/10a) 2.The overall power generation of the basin would be reduced by 4 %–6 % compared to the baseline period. 3. The runoff will decrease during the rainy season and increase during the dry season. 4. The reduction in future runoff will lead to a downturn in hydropower generation that relies on natural runoff.
Europe	[48]	Impacts of climate change on future water availability for hydropower and public water supply in Wales, UK	SWAT	RCP8.5	1. An increase in the frequency of situations in which there is not enough streamflow to satisfy PWS demand. 2. A drop in the total annual volume of water taken out of the ground results in a diminished generation potential. 3.The rate of change will be most pronounced in the medium term (2021–2054).
South America	[49]	Climate change may impair electricity generation and economic viability of future Amazon hydropower	25 GCMs from CMIP5	RCPs 4.5 and 8.5	1. CC scenarios show basin-wide reductions of river discharge (means, 13 and 16 %, respectively) and hydropower generation (19 and 27 %). 2. CC will cause more frequent low-discharge interruption of hydropower generation and less frequent full-capacity operation.
Africa	[50]	Modeling climate change impact on inflow and hydropower generation of Nangbeto dam in West Africa using multi-model CORDEX ensemble and ensemble machine learning	Coordinated Regional climate Downscaling Experiment (CORDEX)	RCPs 4.5 and 8.5	1.At the annual, monthly and seasonal time scales, the inflow and energy simulated over the future periods (2020–2039, 2040–2059, 2060–2079, and 2080–2099) will decrease relative to the historical period (1986–2005) for both RCPs in the range of (2.5–20.5 % and 1–8.5 % for inflow and energy, respectively)
South and Central Asia	[51]	Projected increase in hydropower production in India under climate change	Variable Infiltration Capacity (VIC) model.	RCP 8.5	1. All the hydropower projects are projected to experience a warmer and wetter climate in thefuture. 2. Multimodel ensemble mean annual average temperature (precipitation) is projected to riseup to 6.3 ± 1.6 °C (18 ± 14.6 %) in the catchments upstream of the other reservoirs by the end of the21st century under RCP 8.5. 3.Mean annual streamflow (up to +45 %) and hydropower (up to +25 %) production isprojected to rise under the future climate.
	[52]	Towards climate-adaptive development of small hydropower projects in Himalaya: A multi-model assessment in upper Beas basin.	The Water Evaluation and Planning (WEAP) model	RCPs 4.5 and 8.5.	1. Projected the total streamflow to have widespread uncertainty in the magnitude but shows noticeable changes in the seasonality. 2. One of the SHPs utilizing high flows with low hydraulic head shows power generation behaviour similar to streamflow projections. 3. The annual hydropower production is projected to change by 2–21 % (RCP4.5) and −5 to 40 % (RCP8.5) by the end of the century. 4. The second plant that uses lesser flows but high head maintains its designed power production consistently throughout the century.

**Table 2:** A Summary of Impactof CCon streamflow.

**Regions**	Reference	Main Purpose of the study	Method/Model Used	Climate Scenario/GCM	Main Outcomes:
**North and Central America**	[53]	Quantifying the relationship between streamflow and climate change in a small basin under future scenarios	SWAT	Projected climate data from two GCMs under RCPs 4.5 and 8.5	1. Shifts in the amount of forestland by 2030 (−21.07 km^2^) and the amount of land devoted to intensive urban use (+5.4 km^2^). 2. A general downward trend in runoff for both of the selected GCMs, when the RCPs are set to 4.5 and 8.5. 3. It's possible that precipitation makes up a larger portion of the RC of streamflow.
	[54]	Projecting impacts of wildfire and climate change on streamflow, sediment, and organic carbon yields in a forested watershed	SWAT-OCSM	Not available	1.CC scenarios indicated lower streamflow relative to the baseline period (1995–2014), with 25.3–46.9 % less water in the near future (2015–2034) compared to 9.9–31.8 % less water in the distant future (2043–2062). 2.Sediment concentrations generally decreased, whereas TOC concentrations increased, in both the near future and distant future scenarios 3.Wildfire simulations compounded with CC changing local hydrology, increasing surface runoff, sediment, and TOC transport by over 500 % in some sub-catchments. 4.Sediment yields only increased up to 6.5 % and TOC yields increased up to 13.1 % at the watershed outlet
**East Asia Pacific**	[55]	Sensitivity of streamflow patterns to river regulation and climate change and its implications for ecological and environmental management.	Generalized Additive Model (GAM), Path Analysis (Structural Equation Model).		1.Low flows and medium flows increased by 26 % 2.High flows and overbank flows decreased by 31 % during the period between 1977 and 2018. 3.Current River regulation and flow diversion practices would dominate future change in magnitude, duration, and frequency of the streamflow 4.The timing of flow metrics would be dominated by variation in rainfall.
	[56]	Assessment of climate change impacts on the streamflow for the Mun River in the Mekong Basin, Southeast Asia	SWAT	34 GCMs under RCP2.6, RCP4.5, and RCP8.5	1.A rise in temperature across the board for all RCPs. 2.The three possible futures all predict a decrease in the annual average precipitation 3. An increase in the volume of flow in the stream
	[57]	Future climate change impacts on streamflow and nitrogen exports based on CMIP5 projection in the Miyun Reservoir Basin	SWAT	RCPs 4.5 and 8.5	1.The outcome shows that the MRB would be warmer and more humid. 2.The ensemble mean changes in average annual precipitation are expected to be more than 5.4 % during the period of 2021–2035 and 12.5 % during 2051–2065. 3.Future streamflow and TN loading projection are expected to increase in two future periods. 4.Changes in streamflow and TN loading would be higher in summer than in other seasons. 5.The uncertainty ranges in TN loading projection is larger than that in streamflow projection. 6.The probability that streamflow and TN loading increase would be higher in the period of 2021–2035 than in 2051–2065.
	[58]	Seasonal differences in future climate and streamflow variation in a watershed of Northern China	Regional Hydro-Ecological Simulation System (RHESSys) GFDL-CM3, CanESM2, CNRM-CM5	RCPs 8.5, 4.5, and 2.6	1.Future climate shows a drier and warmer drift in the summer monsoon period contrasting with other seasons in the watershed. 2.Precipitation will decrease by 47.5–57.2 mm throughout the summer monsoon period though increasing annually. 3.Future summer streamflow will reduce accordingly, which is also driven by increased evapotranspiration owing to increasing temperature. 4.An increased dispersion coefficient of streamflow also indicates more dramatic variations in summer. 5.The degree of streamflow will reduce with the extended return periods (*p* < 0.01).
	[59]	Impacts of changes in climate and land use/land cover under IPCC RCP scenarios on streamflow in the Hoeya River Basin, Korea	SWAT	RCP 4.5 and RCP 8.5	1. Changes in LULC are less significant than those brought on by climate change. 2. The effects of the LULC change on the streamflow were very significant.
	[60]	Assessment of the impact of climate change and mining activities on streamflow and selected metal's loading in the Chindwin River, Myanmar	SHETRAN hydrological model	1.ACCESS1-CSIRO-CCAM, 2. CCSM4-CSIRO-CCAM, 3. CNRM-CM5-CSIRO-CCAM	1.It is anticipated that future discharges will decrease by between 3.4 % and 36.5 % across all stations with the exception of Mokekalae station as a result of climate change. 2. The anticipated metal loading in the future climate conditions exhibits a decreasing pattern that is analogous to the anticipated discharge pattern.
	[61]	Impacts of climate change and reservoir operation on streamflow and flood characteristics in the Lancang-Mekong River Basin	Variable Infiltration Capacity (VIC) model	No data	1. The reservoirs had a significant impact on the amount of water flowing through the stream. 2. A 5 % decrease in the streamflow that occurs on an annual average. 3.The operation of the upstream reservoir had a significant impact on the streamflow, especially when compared to the baseline period of 1985–2007. 4.As a result of climate change, the intensity of the flood and the frequency with which it occurred both increased by up to 14 % and 45 %, respectively.
	[62]	Probable streamflow changes and its associated risk to the water resources of Abuan watershed, the Philippines caused by climate change and land-use changes	SWAT	GCM under RCP4.5, and RCP8.5	1.LULC was the cause of a reduction in the amount of grassland and forest cover. 2. The combined effects of CC and LULC led to a reduction in the amount of streamflow and precipitation. 3. The combined effects of CC and LULC led to a reduction in ET and in groundwater recharge.
**Europe**	[63]	Analysis of the occurrence, robustness and characteristics of abrupt changes in streamflow time series under future climate change.	SWAT	No data	1.The outcome indicates that the occurrence of change points due to CC is spread throughout the decades, but strongly clustered in the current and upcoming decade. 2.The highest significant alteration of IHA occurs in the lowland-, less in the alpine- and least in the mid-range mountain catchment. 3.The transition of hydrologic regimes to an alternative state after the identified change points is likely to occur because the majority of indicators move out of the current state of RVA. 4.Potential future hydrological changes both in timing and severity provides an early warning signal of changes for climate change mitigation.
**Africa**	[64]	Climate change and the response of streamflow of watersheds under the high emission scenario in Lake Tana sub-basin, upper Blue Nile basin, Ethiopia.	Soil Water Assessment Tool (SWAT)	RCP8.5	1.Change in maximum temperature ranges from 2.93 °C (November) and 5.17 °C (March), and the change in minimum temperature also ranges from 3.08 °C to 4.79 °C on a monthly basis. 2.Projected rainfall to increase up to 29.75 % (November) and reduce up to 9.26 % (March) in diverse seasons. 3.The flow is predicted to rise up to 27.82 %, 27.47 %, 26.47 %, and 24.97 % in Ribb, GilgelAbay, Gumara, and Megech watersheds, respectively, and it is also decrease in winter and spring seasons. 4.Averagely, the streamflow is anticipated to increase by 5.89 %, 5.63 %, 4.92 %, and 4.87 % in Ribb, Gumara, Megech, and GilgelAbay watersheds, respectively.
	[65]	Climate change impact on streamflow in a tropical basin of Ghana, West Africa	SWAT	GCM under RCP4.5, and RCP8.5	1.An increase in the annual streamflow based on the RCP8.5 scenario 2. A reduction in the average yearly streamflow under RCP4.5 3.The monthly streamflow ranged from −15 % to 23 % when RCP4.5 was used. 4. Under the RCP8.5 scenario, the monthly streamflow ranged from −24 % to 24 %.
	[66]	Impact of climate change on future precipitation amounts, seasonal distribution, and streamflow in the Omo-Gibe basin, Ethiopia	SWAT	Projected climate data from two GCMs under RCP4.5 and RCP8.5	1. An increase in temperature, but a notable drop in both the amount of precipitation and the streamflow. 2. The temperature will rise between 2.40 and 3.340° Celsius (RCP 4.5) and between 2.6 and 4.540° Celsius (RCP 8.5) 3. The amount of precipitation falls between 10.77 and 13.11 % under RCP 4.5, and 11.10–13.86 % under RCP 8.5

**Table 3:** A Summary of Impact of CC and LULC changes on hydropower generation and development.

Regions	Reference	Main Purpose of the study	Major Criteria considered	Method/Model Used	Climate Scenario/GCM	Highlights/Results
**North and Central America**	[67]	Evaluating the joint effects of climate and land use change on runoff and pollutant loading in a rapidly developing watershed	No data	Storm Water Manage-ment Model (SWMM)	RCPs 4.5 and 8.5	1. It is anticipated that changes in future LULC will have a greater impact on runoff, total suspended solids, total nitrogen, and total phosphorus than the projected changes in climate. 2. The combined effects of climate and LULC change are likely to generate increased runoff, as well as increased loading of nutrients and sediment.
	[68]	Climate Change, Land Use/Land Cover Change, and Population Growth as Drivers of Groundwater Depletion in the Central Valleys, Oaxaca, Mexico	Climatological data, DEM, Hydrological data	GFDL-CM3 and HELP Model	RCPs 4.5 and 8.5	1. The region's mean annual temperature has increased 1.79 °C and urban areas have increased at a rate of 2.3 km^2^/year. 2.Population growth has increased water consumption by 97.93 × 106 m^3^/year. 3.The volume of groundwater is shrinks at a rate of 284.34 × 106 m^3^/year
	[69]	Climate and land cover change impacts on stormwater runoff in large-scale coastal-urban environments	DEMs, Soil data, Spatial variation of land cover, Streamflow data from 14 stations	EPA Storm Water Manage-mentModel 5.1	RCPs 4.5 and 8.5 scenarios.	1. Coastal-urban environments had the most significant influence on the climatic factors that determined the runoff changes. 2. CC and land cover that occurred at the same time caused synergistic effects on runoff. 3. Anticipation ofa significant increases of up to 118 % in coastal urban runoff by 2050s and 2080s 4. Greater increases in the potential for flooding and runoff have been observed in urban centers.
**East Asia Pacific**	[70]	How climate change and land-use evolution relates to the non-point source pollution in a typical watershed of China	DEM, Land use, Soil data, Weather data Runoff, NH3–N, Meteorologicaldata	SWAT model	No data	1. The effects of climate change resulted in a reduction of all nutrient losses. 2. The influence of weather is substantially greater than that of land use, with values hovering around 90 %. 3. The impact of land use has a beneficial accumulative effect over the course of decades.
	[71]	Impacts of climate and land use change on groundwater recharge under shared socioeconomic pathways: A case of Siem Reap, Cambodia	**Spatial Data** Land Use Maps, DEM, Soil Type Map, Road Network Map, Population Density Map, Restricted Area Maps **Hydro-meteorological Data** Observed Rainfall, Temperature, Water Level, GCM Climate datasets	CMIP6, GCMs, DynaCLUE land-use and SWATmodel	SSP2-4.5 and SSP5-8.5.	1. An overall rise in the amount of precipitation predicted by both SSPs 2. It is likely that the maximum annual temperature will rise by 0.024 °C each year, and by 0.049 °C each year under SSP2-4.5 and SSP5-8.5, respectively. 3. An increase in minimum temperature that is relatively higher than the average is anticipated. 4. The analysis of past changes in land use revealed a 373% increase in the size of urban settlements between the years 2004 and 2019. 5. The rate of expansion will increase from 2019 by 55 % in 2030, 209 % in 2060, and 369 % in 2090 under the SSP2
**South and Central Asia**	[72]	Investigating the impact of climate and land-use land cover changes on hydrological predictions over the Krishna River basin under present and future scenarios	DEM, LULC map, soil map, Rainfall, streamflow, minimum and maximum temperature	SWAT	RCP 4.5 and RCP 8.5	1.The combined impact of climatic and LULC results in shifts on the water balance, along with multimodel projections. 2. An emphasis was on the significance of LULC changes and water storage structures in relation to the performance and simulations of the model.
	[73]	The Impact of Climate Change as Well as Land-Use and Land-Cover Changes on Water Yield Services in Haraz Basin	DEM, soil data, climate data, plant available water content (PAWC)	InVEST Model;	No data	1. Annual precipitation showed decreasing amounts in the three studied years from the northwest to the south. 2.Water yield reduced during the study period 1992–2007 and 2007–2016, and climate change plays a vital role 3. Rainfall will recover in 2026 and water yield will increase in the northern sub-basins. 4. In the long run (1992–2026), the contribution of LULCC and CC factors to water yield are equal.
	[74]	Alteration of groundwater recharge areas due to land use/cover change in Kathmandu Valley, Nepal	DEM, geology, climate change data, socio-economic data	CLUE-S, SWAT	RCPs 4.5 and 8.5	1.The modeling results show that the river runoff for RCP4.5 scenarios is projected to increase by 37 %, 21 %, and 12 %, respectively, for climate change only, LULC only, and integrated changes of both. 2. LULC change resulted in an increase in average annual flow, however, a decrease in base-flow.
**Africa**	[75]	Assessment of Spatio-Temporal Changes of Land Use and Land Cover over South-Western African Basins and Their Relations with Variations of Discharges	LULC Data Sources Precipitation and Discharge Data	Mann–Kendall test	N/A	1.All the studied basins present an increase in water bodies and LULC classes 2.Increases in water bodies and LU are as a result of an increase in small reservoirs of dugouts and dam constructions. 3.Statistical analysis shows a variation between precipitation and discharge data at the three basins. 4.The variation in discharge is primarily due to LULC changes. 5.The hydrological modification of the LULC changes, the climate of the region, water availability and quality and hydropower generation may be impacted by these changes in land surfaces conditions.
	[76]	Modeling projected impacts of climate and land use/land cover changes on hydrological responses in the Lake Tana Basin, upper Blue Nile River Basin, Ethiopia	DEM, soil data, LULC data, Meteorological data, Hydrological data, GCMs	SWAT& IPEAT (Integrated Parameter Estimation and Uncertainty Analysis Tool)	RCP2.6, RCP 4.5 and RCP 8.5	1.The result of a climate prediction suggests an increase in both the maximum and the minimum temperature. 2.The basin could see an increase in precipitation of up to 25%. 3.An increase in ET by up to 0.84 %, 59.8 %, and 55.5 % under LULC, climate, and combined climate and LULC change 4.The magnitude of the shift in emissions under RCP8.5 is greater than that under RCP2.6 and RCP4.5.
	[77]	Climate, Land Use and Land Cover Changes in the Bandama Basin (Côte D'Ivoire, West Africa) and Incidences on Hydropower Production of the Kossou Dam	LULC Data Hydroclimatic and Energy Production Data	Kriging method, Mann–Kendall test	N/A	1.The outcome reveal that water bodies areas and LU have increased by 1.89 %/year and 11.56 %/year respectively 2.Streamflow presents greater change in magnitude compared to rainfall 3.Streamflow varies greatly than the rainfall does annually except during driest months possibly due to LULC change. 4.Kossou hydropower generation is significantly decreasing (p-value 0.007) at both monthly and annual scales possibly due to water abstraction at upstream
	[78]	Land Use and Land Cover Changes under Climate Uncertainty: Modelling the Impacts on Hydropower Production in Western Africa	Land Use and Land Cover Change data Rainfall and Stream Flow data	WEAP Model	No data	1.Historical rainfall analysis and stream flow changes shows variability in the annual coefficient of rainfall and stream flow as 8.6 % and 60.85 % respectively. 2.The LULC analysis shows important variations in vegetative areas and water bodies. 3.The WEAP model evaluation showed that combined effects of LULC and climate change reduce water availability for all of demand sectors, including hydropower generation at the Bui hydropower plant. 4.Projections are that the Bui power production will increase by 40.7 % and 24.93 % under wet and adaptation conditions respectively 5.Bui power production will decrease by 46 % and 2.5 % under dry and current conditions respectively.
	[79]	Assessing the Effect of Land/Use Land Cover and Climate Change on Water Yield and Groundwater Recharge in East African Rift Valley using Integrated Model	DEM, LULC, Soil data, Weather data, Hydrological data (at Meki) GCM3(CMCC-CM) GCM2 (MRI-CGCM3) GCM1 (bcc-csm1-1-m) Observed climate	SWAT - MODFL	No data	1. The majority of the LULC consists of cultivated land, with an additional 5 % spread across the areas of forest and grassland. 2. Anticipation of an 8–11 % increase in the average temperature and a 3–6% increase in the average precipitation. 3. The change in climate has a significant impact on the spatiotemporal distribution of WYGR, in contrast to the effect that LULC change has, which is negligible. 4. A decrease in water yield of up to 48 % and the transition of perennial rivers into intermittent rivers are anticipated.
**South America**	[80]	Disentangling the historic and future impacts of land use changes and climate variability on the hydrology of a mountain region in Brazil	DEM Land use Soil Weather data Hydrological data	SWAT model	RCP 8.5	1.The primary forces that have historically influenced the movement of water in this region are shifts in the patterns of climate. 2.The expansion of forested land in the Green Road scenario will bring about a reduction in surface runoff, which will, in turn, bring about an increase in water yield.
	[81]	Land use change scenarios and their effects on hydropower energy in the Amazon	DEM Land use Soil Weather data Hydrological data	SWAT model	No data	1.LULCC have a significant influence on the basin's overall water balance due to their effects. 2. The response of the Tucuru hydropower system to various land use change scenarios is inefficient with regard to energy use.
	[82]	Public policies on water resource management and its impacts on the context of climatic changes and alterations in land use and land cover in small and protected rainforest river basins	DEM, Land use, Soil data, Weather data Runoff, NH3–N, Meteorologicaldata	SWAT - MODFL	RCPs 2.6, 4.5, 6.0, and 8.5	1.The estimated monthly average flow reduces by the end of the century. 2. The forecast change in LULC impacted on the decreased average monthly flow to 2.9 % by 2099 (RCP 2.6), 2.5 % (RCP 4.5), and 2.7 % (RCP 6.0 and 8.5). 3.The evapotranspiration will be less than 2.4 % (RCPs 2.6, 6.0) and 2.3 % (RCPs 4.5, 8.5) 4.Local economic development would be interfered due to the faulty water resource maintenance of the basin.

## 2 literature review method

A systematic literature review (SLR) was carried out on the impact CC and LULC has on the generation and development of hydropower. The review also includes impact of CC on the streamflow. According to Ref. [83], the SLR summarises and synthesizes the conclusions of current study literature on a given topic or area. This affords the opportunity to unearth important concepts and fill up research gaps, and types of proof that can impact practice as well as policymaking. On the basis of [84], we utilised the Preferred Reporting Items for Systematic Reviews and Meta-Analyses (PRISMA). Following the definition of SLR provided by Ref. [83], we began this review by examining references that alluded to the impact of CC and LULC on the generation and development of hydropower, with a particular focus on articles that were published in the most recent decade (post-2010). Only articles, whose findings were, based on the “impact of CC on hydropower”, “impact of CC on streamflow”, and “combined impacts of CC and LULC on hydropower”, were included in the analysis. This was done to eliminate any potential bias towards a specific author or region. The flow diagram for the selection of studies using PRISMA is shown in Fig. 2.

For the purpose of this review, we looked at a total of one hundred and fifty-eight (158) erudite articles, which were published in English language and in reputable journals between the years 2010and 2022. Although every effort was made not to include conference proceedings, this review made sure to take into account the keywords that were outlined in its primary purpose.

### 2.1 summary of literature selection

**Fig. 3:**
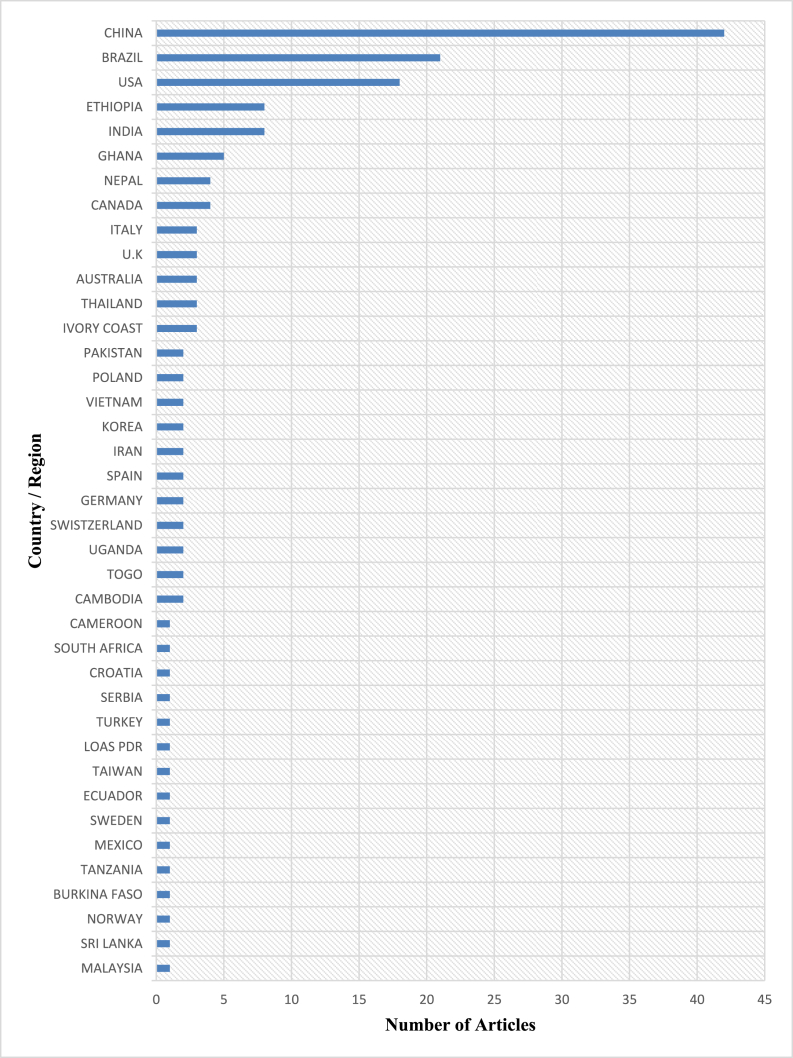
Countries of study.

**Fig. 4:**
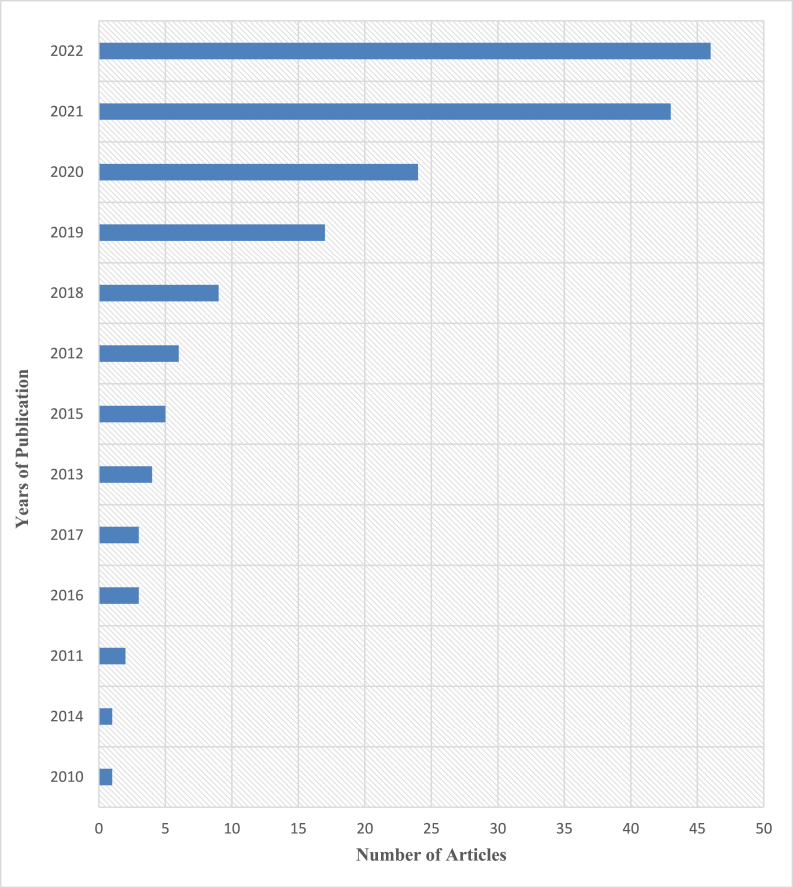
Years of publication (2010–2022).

**Fig. 5:**
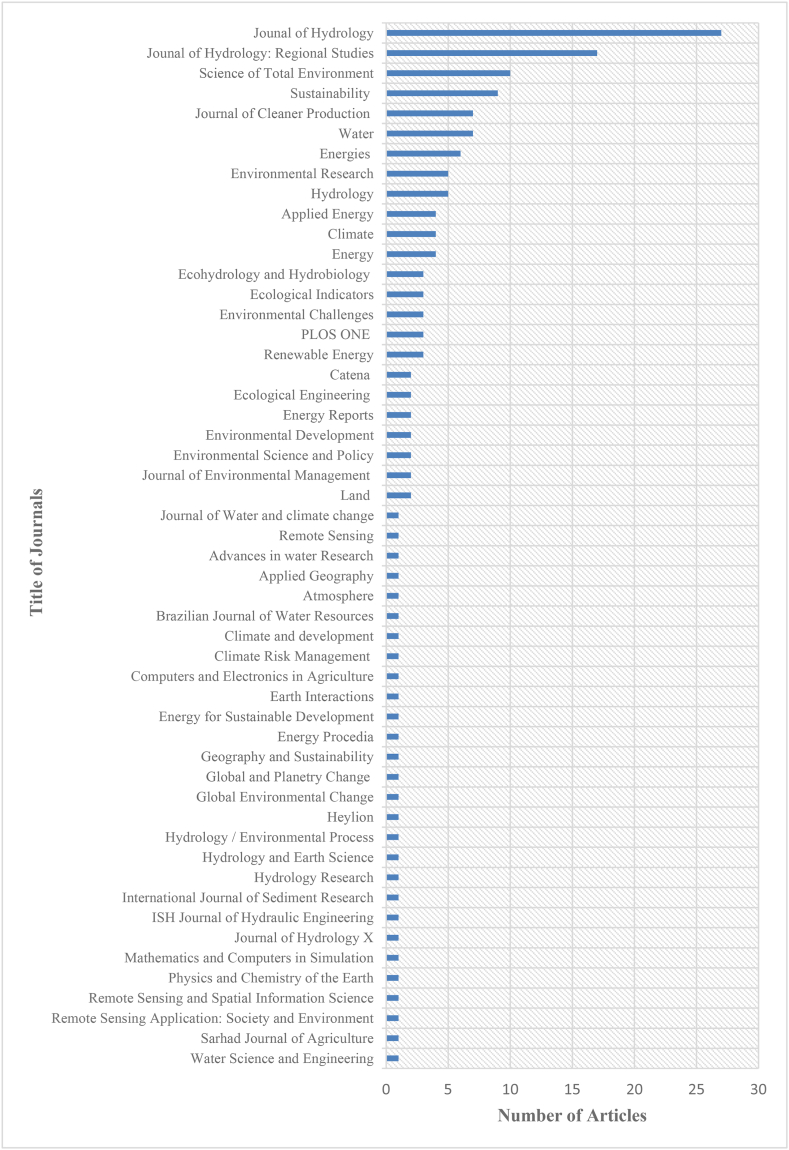
Number of publications in the Journals reviewed from 2010 to 2022 for this study.

The databases of Scopus, Web of Science, and Google Scholar were searched, and the results initially yielded a total of 25,711 articles. There were 24,489 articles that were removed from the database because their full texts were not available online or they were not pertinent to the topic that was being investigated or they were duplicates. Following an additional screening, a total of 1064 articles were removed from consideration because they did not conform to the goals that had been established for this review. In the end, it collected 158 articles for further examination from a variety of countries of study, publication years, and academic journals. Fig. 3, Fig. 4, Fig. 5 provide a summary of the data that was collected.

Only articles that connected CC to the generation and development of hydropower, the impact of CC on streamflow, and the individual or collective impacts of CC and LULC on hydropower were considered for this review. This was done in order to stay consistent with the objectives that were set for this review. The summary of the screening in the form of a flow chart is in Fig. 1. It does so in a way that is consistent with the definition of PRISMA.

As shown inFig. 3, Ghana is among the top six countries that have produced papers for this review. Thus, five (5) publications were examined from Ghana whilst eight (8) were examined from India and Ethiopia. USA, Brazil and China produced 14, 21 and 42 respectively. Fig. 4 displays the complete list of countries from which journals were collected for this study's analysis. Between 2010 and 2022, the sum of published literature onCC and LULC on the generation and development of hydropower saw an increase as displayed in Fig. 4. Fig. 5 displays the complete list of Journals that were accessed, and the number of articles retrieved from each journal.

### 2.2 Summary and development of a catalog

In all, the articles comprising this review came from a total of thirty-nine (39) different countries in the six regions of the world.These regions includeAfrica, Europe, East Asia Pacific, South and Central Asia, North and Central America as well as SouthAmerica. The regions contributed to the following proportions: 12, 11, 9, 5, 3, and 2, respectively to the study.

Between 2010 and 2022, the sum of published literature on the impact ofCC and LULC changeson the generation and development of hydropower saw an increase as displays in Fig. 4. Fig. 4thus displays thecomplete list of years from which journals were collected for this study's analysis. Fig. 5 displays the complete list of Journals that were accessed, and the number of articles retrieved from each journal.

**Fig. 6:**
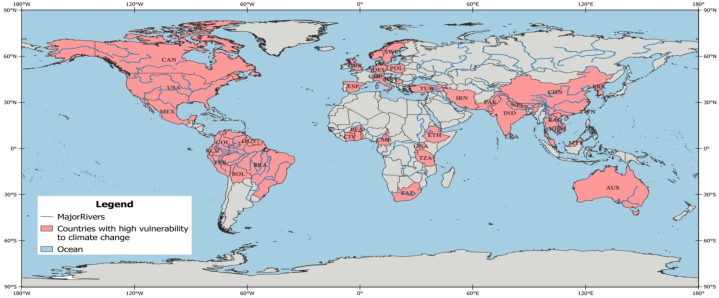
Relevant regions to the discussion of risks to hydropower from CC. LULC.

The map below (Fig. 6)clearly highlights all regions that are likely to experience changes in the future due to CC, LULC, and the combination of CC and LULC.

Over the course of the 12-year period (2010–2022) under consideration, a total of 52 journals were investigated. Every one of the articles was published in a journal that specialised in hydrology, ecology, the environment, remote sensing, sustainability, water, applied geography, or one of those subjects' related subfields. The catalog of journals that were accessed is presented in Fig. 3, along with the total sum of articles read from every publication.

## 3 RESULTS and DISCUSSIONS

### 3.1 Impacts of CC on generation and hydropower development

Significant impacts of a CC on water resources, in turn, have repercussions for the generation of hydroelectric power. [101], therefore linked the generation of hydroelectric power to the geographical hydrological state and response of a catchment and its reaction to how sensitively it reacts to changes in the quantity of water and the seasons. Different researchers have attributed CC to human activities (e.g., changes in land use) as the primary drivers responsible for modifying hydrological processes [[102], [103], [104]]. The IPCC issued a report in 2013 that forecast how CC would affect water resources across a large portion of the tropics and subtropics. Their findings were in agreement with [105] who also stated that CC is responsible for global water shortages. Also [ [42,106]],stated that CC is responsible for the increase in the severity of extreme rainstorms and extreme floods. Furthermore [ [104,105]], believethat CCcan have a significant impact on hydropower plants, which can lead to a decrease in the amount of power produced by hydroelectric dams.

Quite a lot of studies on CC impact on hydropower have been undertaken in the past years with varied or similar results. For example [33], conducted an analysis of the shifts that have taken place in the generation of hydroelectricity worldwide. Their findings suggest that there are significant differences in hydropower generation between and also within regions [86]. conducted a statisticalanalysis to determine the effects of the factors that influence CC on the circulation of the Prut River. The yearly, seasonal and monthly averages, as well as the trends, of the meteorological and hydrological variables for each period were compared. It was found that there was an increase in the yearly average, highest, and lowest temperatures in the region by 1.04^0C^, correspondingly [87]. estimated the rise of floods and evaporation as the temperature keeps rising, which will result in a decrease in the amount of energy that is produced at the Nam NgumRiver.

A section of researchers worldwide is presently employing the Coupled Model Intercomparison Project (CMIP) of the IPCC in order to investigate the pattern and progression regarding runoff and rainfall in relation to various CC scenarios. For instance, both [ [30,37]] used CMIP5 data to evaluate the performance of the hydropower plants and system respectively.

Other equally important models and different CC scenarios have been employed in evaluating the influence of CC on hydropower generation throughout the body of academic research. For instance, in assessing the impact of CC on hydropower [38], employed the Water Evaluation and Planning (WEAP) model [39], used the MHD-INPE hydrological model and [40] utilised a simulation model.

### 3.2 Impacts of CC on streamflow

There is a rising demand for water resources all over the world to satisfy the ever-increasing requirements for irrigation, as well as for residential and commercial uses. In research conducted by Ref. [107], it was estimated that more than half (52 %) of the world's population would face severe water crises by the year 2050 if there is no intervention. Already, research conducted by Ref. [108] has discovered that approximately 36 % of the world's population that resides in developing countries is affected by water scarcity. According to Ref. [109], there is a need for accurate forecasting of streamflow so as to have optimal operation of reservoir systems and efficient operation of hydropower stations. The accurate forecasting helps decision-makers make the management decision to reduce the effects of the flood on both humans and structures [ [110,111]]. LULC, in addition to climate variability, has an influence on streamflow. Previous studies have shown that a reduction in the amount of vegetation and the infiltration capacity of the soil causes an increase in the amount of streamflow [ [112,113]].

The research conducted by Ref. [114] demonstrated that the observed decadal changes in the streamflow were caused primarily by anthropogenic activities, despite the fact that changes in precipitation also made some decadal contributions. At the Yellow River Basin in China [115], found that after the 1980s, there was a significant drop in all of the streamflow signatures measured at the four stations, particularly for high-flow magnitudes. Comparing the consequences of land-use change and CC [116], discovered that climate change had a more pronounced impact on streamflow, with a share of 78.8–98.8 %. The Mann–Kendall test for sudden change and voltage level slopes was utilised by Ref. [117] in order to investigate the impact of anthropogenic activities in addition to CC on streamflow. Three key turning points and rapid changes in streamflow occurred in 1963, 1983, and 1991, according to their findings. It was determined that these observations are congruent with worldwide ENSO episodes and volcanic activities.

[118] investigated the impact that CC has had on the complexity of stream-flow in the headwater region of the northeastern Tibetan Plateau over a number of periods. They arrived at the conclusion that the Upper Heihe River (UHR) watershed has witnessed a large increase in the amount of complexity in its streamflow since 1972 as a direct result of CC. Additionally, the influence of precipitation on the complexity of the streamflow was found to have decreased, while the influence of air temperature was found to have grown. The research conducted by Ref. [119] on the effects of climate change on the streamflow of the Brahmaputra River. The result of changes in snowpack attributes shows that the basin's annual streamflow is growing while, at the same time, the reliability of the water supply is decreasing. This is as a result of the increased spill volume resulting from greater snowmelt concentration during the wet period and partly as a result of the increase in evaporation losses that are occurring as a result of higher temperatures.

The General Circulation Model (GCM) was utilised by a number of researchers across the globe in order to make projections regarding the effects that CC will have on streamflow as well as the generation of hydropower. For instance Refs. [34,[120], [121], [122], [123]], use GCMs to quantify the effects of CC on streamflow as well as hydropower generation. These studies were published in various academic journals.

The influence that alterations in climate have on the flow of water all over the world has been studied using a variety of methods, including those listed here. For example [124], evaluated the effects of climate change on hydrologic systems by developing a SWAT model-based analysis workflow. The authors found that theHargreaves method was more accurate than the Penman-Monteith method. The annual mean runoff (Qm) and annual maximum runoff (Qp) will increase by 1.8 % and 2.6 % in the 2040s, as well as 14.7 % and 18.6 % in the 2080s. Arc SWAT was the model that [125] utilised in order to evaluate the possible effects that CC could have on the seasonal and annual streamflow of the Nicolet River (Southern Quebec). The data that was projected for the future climate showed that there would be increases in both the average temperature (+2.5 °C) and the amount of precipitation (+21 %).

Using a distributed hydrological model and statistical methods [126], quantified the streamflow decline in the upper Yangtze River (UYR) and the impacts of CC and human activities from 1961 to 2015. According to the findings of the study, the contributions of CC to human water consumption, reservoir impoundment, human-induced vegetation change, and the decline of streamflow are as follows: 62.5 %, 19.7 %, 18.4 %, and 1.8 %, respectively.

### 3.3 Impact of CC and LULC on the generation and development of hydropower

Both human activities and changes in climate are the primary factors that have effect on hydrologic processes. Land use and land cover (LULC) activities are one of the main causes of these changes. According to Ref. [127], the influences manifest themselves as percolation, infiltration, groundwater, and runoff. The processes of interception, surface streamflow, and groundwater replenishment are all kept in equilibrium by changes in land use [ [128,129]]. According to Refs. [[130], [131], [132]], CC and LULC are the two most important influencing factors in the hydrological cycle that can affect the accuracy of parameters. These factors can have an effect on how water moves through the system. In research conducted by[133,134] they stated that CC directly impacts regional precipitation, temperature, and humidity. As a result of that it affects components of the water and energy cycles. In the case of LULC changes, its impact alters the partitioning of precipitation into evaporation, transpiration, runoff, interception, and infiltration. This in turn affects soil moisture contents and energy cycle [135]. Other researchers such as[112,113,136] suggest that an increase in streamflow can be attributed to a reduction in the amount of vegetation as well as the capacity of the soil to infiltrate water. According to Ref. [137], other variables that influence hydrologic processes include precipitation, ET, solar radiation, temperature, soil type, topography and LULC. In addition [113], found that potential evapotranspiration (PET) has an effect on water resources via the indirect effects that are caused by wind, temperature, and solar radiation [138]. also found that the water yield of each LULC scenario under RCP4.5 had a decreasing trend. According to the findings, changes in LULC had a greater impact on water purification than CC did on water yield. They concluded that CC had a greater impact on water quality.

Within the period of our analysis, different hydrological models have been widely used for simulating hydrological processes and responses at various scales and regions. These models, which use input parameters and refined mathematical models to simplify the natural hydrological processes, have been used by a large number of researchers [[139], [140], [141], [142]]. The regional climate models (RCMs) under RCPs 4.5 and 8.5, in addition to the WEAP tool was also used by Ref. [143] to study the combined effects of CC, LULCC. They concluded that climate variables had an effect on the simulated water accessibility for a variety of water demand sectors.

The Soil and Water Assessment Tool, also known as SWAT, has been put to extensive use in a number of studies that have been published in recent years Ref [e.g. [[81], [88], [144], [145], [146], [147]]]. In another instance [148], made a prognostication regarding the watershed hydrological response to CC and LULC changes in Highland Ethiopia. All four RCMs used, predicted a decrease in future precipitation and an increase in future temperature under RCPs 4.5 and 8.5. The SWAT model was also utilised by Ref. [149] in order to evaluate the potential effects of future LULC and CC on the streamflow from the Angkor Temple Complex. The result shows a decrease on seasonal and annual timescales in every scenario. Despite the decrease, Angkor Temple Complex will still be operating under the current LULC and climate regime. The assessment of the relationship between LULC and CC was carried out using a combination of SWAT and ArcGIS in research carried out by Ref. [150]. They used a climate scenario of RCP 4.5 with the CCCMA model and a SWAT embedded in ArcGIS to assess the influence of CC and different LULC on the water balance in the Tordzie watershed. They came to the conclusion that LULC had a significant impact on the hydrology of the Tordzie watershed They projected the water balance for the year 2050 to be an increase in surface flow of 19.00 % but a decrease in groundwater of 36.40 %, a base flow of 0.77 %.

In addition to the SWAT model, other studies also made use of a variety of other hydrological models to evaluate changes in LULC, and CC. For instance Ref. [151], evaluated the impacts of CC and LULC on the hydrology of the Ashti Catchment, India. They use the variable infiltration capacity (VIC) model whilst [152] also studied the impact of climate and LULCC on entire India under different assumed plausible hypothetical scenarios using VIC [151]. demonstrates that ET is primarily determined by the different classes of vegetation. They also demonstrate that ET for forest cover is greater than ET for grassland, simply because trees with deeper roots extract soil moisture from the deeper soil layers. The study also emphasised that the hydrological repercussions of CC in the Ashti Catchment over the past 40 years have not been very significant. The research conducted by Ref. [152] demonstrates, despite this, that even slight alterations to the climate can bring about significant shifts in the hydrological cycle and its components.

### 3.4 Strategies to mitigating the impact of CC on hydropower

The impact of CC on hydropower generation and development is quite enormous and cannot be reversed. Also, quite a substantial amount is usually invested in the construction of hydropower. In the light of above, the main concern now is how to reduce the impact of CC on the generation and development of hydropower. Mitigation and adaptation strategies are two CC broad policies that has been identified as possible causes of action that can offer a coordinated reaction to the danger posed by CC [153]. analyzed the adaption possibilities for maximising hydropower production in circumstances of CC by looking at a number of reservoir rule curves and found that modifying the rules that are already in place is one way to mitigate the potentially damaging impacts of global warming. However, the findings of [154] on adaptation strategies to the influence of CC on Brazil's hydroelectric production show that the effects of CC would result in much greater emissions in the absence of CC mitigation legislation and robustness of mitigation techniques in the face of adaptationissues, whereas adaptation strategies are not as effective. Some mitigation pathways that have been identified are.

1. Making more effort to the development of alternative energy sources such as biomass, solar, geothermal, hydro, ocean, wind, etc. Theywould go a long way to help in mitigating CC.
2. Enhancement of energy efficiency in household and industrial usage thereby contributing to the mitigation of energy-related GHG contributions.
3. There is the need to do a thorough hydrological assessment which would take cognizance of local and regional impact of CC projections.
4. Other possible risk factors such as floods, storms, landslides etc should be part of the planning and design of the dam construction.

### 3.5 Key drivers of CC that affects hydropower generation and development

It has been determined that a number of different factors act as drivers or forces behind the changes in climate. [[89], [90], [91]], for instance, indicated that the rise in temperature as well as the rise in precipitation impact the flow rates in freshwater and bring changes to water demand and supply. On the other hand [42], also indicated that variations in precipitation, temperature rise, increase in evaporation rate, and glacier meltdown are the driving forces behind CC. These floods and droughts have been identified as the driving force behind CC by Refs. [[92], [93], [94], [95], [155]]. The forces or drivers of CC that were discussed earlier have an effect on the generation and development of hydropower in different parts of the world.

**Fig. 7:**
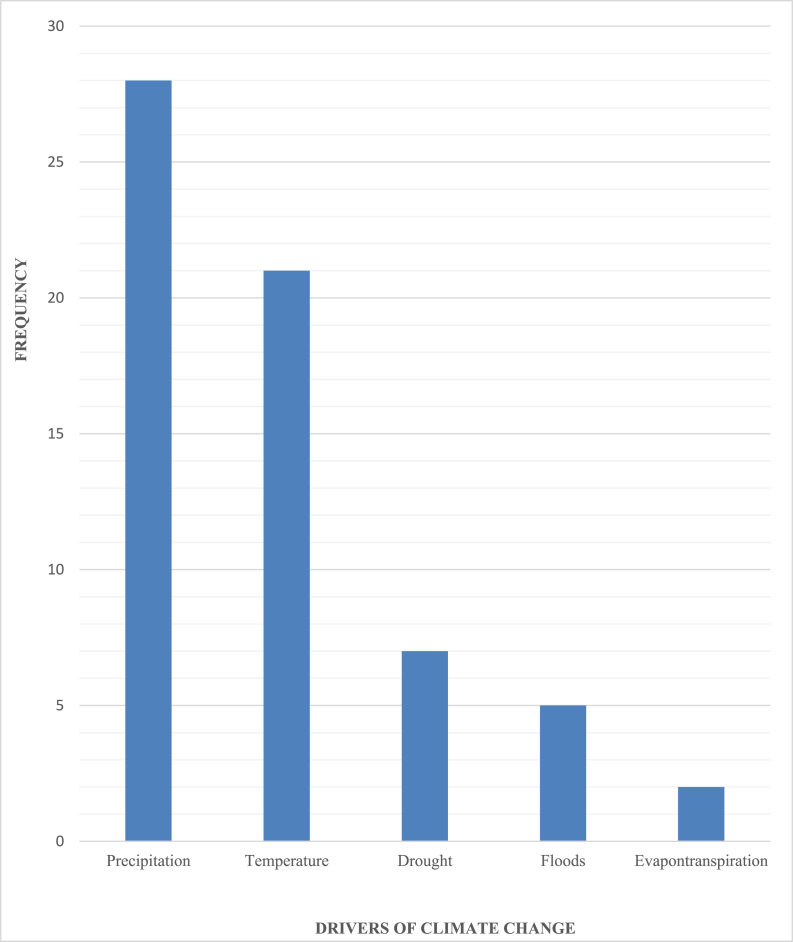
Climate change drivers.

**Source:** [36,42,85,86,87,39,43,44,47,88,[89], [90], [91], [92], [93], [94], [95],[96], [97], [98], [99], [100]]

There are a total of thirty-seven authors who have been identified as attributing the effects of CC to a variety of drivers. These drivers include shifts in temperature and precipitation patterns, as well as floods, droughts, and evapotranspiration. Changes in temperature and changes in the amount of precipitation are the two most important drivers that influence the generation and development of hydropower. These changes are among the four and the outcome can be seen in Fig. 7 below.

### 3.6 Key drivers of LULC that affectsHydropower generation and development

**Fig. 8:**
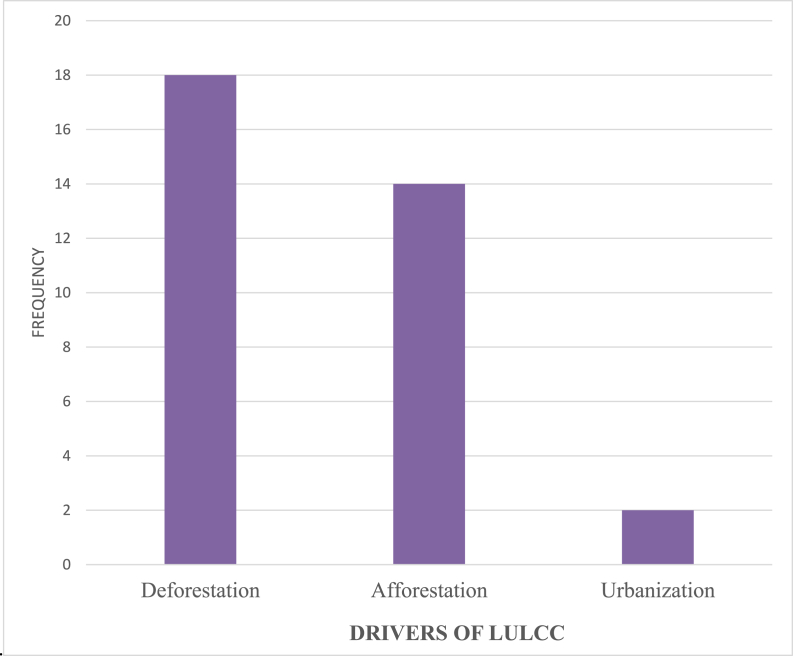
LULCC drivers that affects the generation and development of hydropower.

**Source:** [130,[156], [157], [158], [159], [160], [161], [162], [163], [164], [165], [166], [167], [168], [169], [170], [171]].

Deforestation, reforestation, and urbanization are examples of LULCCs that have a negative impact on the generation and development of hydropower in different parts of the world. Twenty authors in total believe that three distinct factors, including deforestation, urbanization, and afforestation, are responsible for the generation and development of hydropower. For instance Ref. [156], attributed the decrease in yearly flow by up to 15 % and yearly surface runoff by up to 30 % to the rise in forest cover. On the other hand [[157], [158], [159], [160]], attributed the hydrological changes and warming effect to a larger decrease in the ET and variations in the balance between ET, precipitation, and temperature. On the other hand, afforestation and urbanization have been cited as the drivers of LULCC that affect the generation and development of hydropower by Ref. [[161], [162]]. The specific results are broken down into the following Fig. 8categories.

## 4 conclusion

A total of one hundred and fifty - eight (158) papers that were published in a variety of journals were evaluated using the systematic review method. The review focused on recent developments in research as well as the effects of LULC and climate change on the generation of hydropower and its subsequent development. The systematic review unearthed important new information regarding the effects of LULC and climate change on the generation of hydropower as well as the development of new hydropower sources. The effects of climate change are reflected in a decrease in the streamflowas a result of variations in rainfall, a rise in temperature, and an increase in evapotranspiration. These three factors have all contributed to an increase in evaporation and transpiration. Because of this, planners of the largest source of renewable energy in the world need to devise strategies to adapt the generation and development of hydropower to the effects of LULC and changes in climate in order to make the most of the enormous benefits offered by hydropower. The study also found that the increase in temperature and the increase in precipitation are the two primary drivers of climate change. On the other hand, the study found that deforestation and afforestation are the two primary drivers of LULC change, which influence the generation and development of hydropower globally. Additionally, the findings of this study have shown the major drivers of LULC and climate change that are crucial and need to be considered when planning the generation and development of hydropower. These drivers have been revealed as a result of the findings of this study. In conclusion, the findings of this study highlight the fact that the impact of climate change on the generation and development of hydropower is more severe than the impact of LULC changes on the generation and development of hydropower. In comparing the impact of CC to the impact that LULC changes have on the generation and development of hydropower, the impact of CC is twice that of LULC change.

CC coupled with hydrological variability poses a great challenge to the development of hydropower across the globe. It is therefore crucial to have a policy change especially in the face of the new climatic patterns so as to prevent the slowdown of hydropower development. It is also necessary to adopt more effective ways for a climate resilient hydropower.

## Appreciation

The authors would like to applaud the great sacrifices made by editors as well as anonymous reviewers to make this paper what it is today.

## Data availability statement

Data sharing is not applicable to this article as no new data were created or analyzed in this study.

## Declaration of Ai-Assisted technologies In the editing process

During the preparation of this work the corresponding author used Quillbot Premium for language editing. After using this tool, the corresponding authorreviewed and edited the content as needed and takes full responsibility for the content of the publication.

## CRediT authorship contribution statement

**Emmanuel Kekle Ahialey:** Writing – review & editing, Writing – original draft, Methodology, Data curation, Conceptualization. **Amos T. Kabo–Bah:** Supervision. **Samuel Gyamfi:** Supervision.

## Declaration of competing interest

The authors declare that they have no known competing financial interests or personal relationships that could have appeared to influence the work reported in this paper.
